# Filling Teeth

**Published:** 1871-03

**Authors:** E. L. Hunter


### ARTICLE II.

### *Filling Teeth.*

By E. L. Hunter, D. D. S.

*Filling Superior Incisors on their Labial Surfaces.*—

Acidulated buccal secretions having made their ravages upon this part of the teeth, we proceed after careful examination to prepare the cavity in the usual way, viz: by a thorough removal of decay and so shaping it as to permit us to make the mechanical adjustment of the filling firm and lasting. This can be effected, 1st, by mere shape in the general outline of the cavity; 2nd, by lateral grooves in the dentine, and 3d, by one or more retaining points. If soft foil is to be used, the first or second is the method; if adhesive foil or sponge gold, any of the three will answer, provided however, that in certain localities the third method is particularly called for by the termination of one cavity in another, or condition of the surrounding walls, &c. We do not esteem soft foil the most desirable for this locality, as when best condensed it is still softer than a well made adhesive filling, and is much exposed to the wear of the brush. We have often seen these fillings so much worn in this way as to prevent the margins of the cavity to be in their turn countersunk in the same way, thus rendering the loss of much tooth substance necessary in a repetition of the work. If, however, the work should be done with soft foil, we would prepare the gold, Nos. 4 or 6, in carefully folded strips, corresponding to the size of the cavity, i. e. a little smaller than the cavity. These strips should contain no more gold than will permit them to be perfectly flexible under the plugger. We would also prepare a very small strip of No. 20 doubled three times or No. 60 single. Having placed the patient comfortably, and convenient for operating, carefully cleansed the cavity, provided against further moisture by folds of bibulous paper, some other absorbent, or the Rubber Dam, dry the cavity with bibulous paper, flax, cotton, spunk or strip of old linen, and finish if desired by evaporating in the cavity a drop of chloroform.

The strip of soft foil (No. 6.) should now be carried to the cavity and have its end laid on and about the cervical wall, then with a hoe shaped wedge and serrated plugger of sufficient width at the point to thoroughly control the gold, the first fold is carried to the bottom of the cavity and against the cervical wall, the gold being kept in position by the thumb of the left hand. The inner end of each succeeding fold is successively carried in the same direction, and as thoroughly condensed as it is possible to make them by lateral pressure on each fold, being careful to carry them in even, one upon or against another without twisting. When the cavity is one-fourth full, the folds should be played slightly to the right and left to counteract the difference, or make up for the difference between the size of the cavity and the width of the gold. When two-thirds full they may again be placed in as before, and a smaller point of the same kind used except that it might be straighter as more force will be required in carrying the gold to the bottom of the cavity. If the cavity should be a long groove instead of one nearly round, the same rule or mode of operating will apply to it, provided you commence in the one and repeat the same thing in the other, or else in the middle and repeat on each end. Having introduced all the gold possible this way, we open a hole in the centre having parallel sides or walls, with a sharp wedge, and fill with the No. 20 or 60 strip, using the wedge force in the direction there is reason to believe much pressure is needed. Every other point or part of the filling is now tried with a very small point until it is determined that no more gold is required. A sufficient protrusion having been left (and, if possible, no more) for proper condensation, the ends are condensed from the margins towards the centre, first with large and then with smaller points, and finally with very small points with slight serrations and mallet force.

The marks of the plugger having been rubbed out with a dry burnisher, the filling is filed down to near the contour of the tooth, and recondensed with the mallet, reburnished and the patient allowed to free the mouth. The filling may then be filed down, and have the file marks and overlapping scales removed, first with corundum point, and Arkansas stone, then finished with fine pumice stone and precip. chalk, and soft wood, tape or buckskin. After the use of one polishing powder, and before the use of another the filling should be cleansed. After using the chalk, wash, and use the burnisher with soap. Then wipe dry and rub for several minutes with a hank of dry floss silk, which will relieve the brightness and thoroughly remove or test the small overhanging scales, which are always more or less present though not perceptible to the naked eye.

If the margin of the walls of the cavity shall have been made smooth, firm and well defined, we know of no way so effectual with soft foil as the foregoing, as it was but lately that we have been made acquainted with the happy method of "Combining Cylinders and Crystal Gold."

We would also say that in making these soft fillings, we have sometimes varied from the rule as to condensing, first with broad points and then with small, by using the small points entirely, when if too much gold has not been left protruding, the filling will partake much of the nature of adhesive gold, always take a better finish, and be removed from the cavity with extreme difficulty. This is particularly applicable to gold fresh from the beater, when it is slightly adhesive.

We are taught that heavy gold is valuable in filling small cavities, and it was this which suggested to us its use in filling the hole made in the centre of our fillings. We find it can there be carried to the bottom, when by our utmost patience we cannot do so with the light No's, as No's 2, 3, 4, and 5.

Before speaking of the manner of using adhesive gold in the locality just spoken of, we have a word to say in relation to the action of this gold under the plugger, viz: the tendency to draw upon itself, and we do so with a thorough consciousness of our incapacity to treat the matter with the polished science familiar to experienced operators.

Our own views in this matter are given, not because we would set them up in opposition to those of wiser men, but because they were engendered by that experience of which we are writing. It was our opinion that the tendency of the pellet to draw up under the plugger, was an apparent and not a real one, that the more it appeared to do so the less it really did; the rolling up of the pellet being occasioned by pressure upon one point, and not occasioned by a simultaneous pressure of its whole surface. If a coin on an anvil be struck in its centre with a hammer, a concavity will be given to the coin, because its centre is increased without a corresponding increase of its circumference, when if the whole surface receives the blow at the same time, its diameter will be increased without any concavity, without any drawing upon itself. So if large points are used in introducing the gold, this tendency will be obviated to an extent greater as the point approximates in size that of the cavity. If thus caused to adhere to a preceeding pellet which has adjustment against the walls of the cavity, it cannot draw upon itself when it is condensed with smaller points, and being held down must be expanded laterally, when its whole surface shall have been condensed, as gold is malleable. The expansion will be great in accordance with the softnesss and purity of the gold and the pressure made. We are speaking of force in the direction of the bottom of the cavity as a principle. Of course there often arise circumstances which require modifications, as for instance retaining points and undercuts as well as irregularities of various kinds which require to have gold pressed directly into them. Undercuts filled in this way will be forcibly held in position by the expansion of the succeeding gold, as is that packed against the walls, as we are taught, by the succeeding "key stone" in the centre.

In filling the cavity before mentioned, with adhesive gold, we commence by filling first the undercuts or retaining points, if the nature of the case shall have suggested making them, and then with a broad pointed plugger overflow them and extend the mass over the entire floor and against the walls of the cavity. In the introduction of each succeeding pellet of gold, we have endeavored so to place them, that as much or more gold would be near the wall than in the centre; in this way, when there is a base to work upon, build around next the wall higher than in the centre, then work enough in the centre to make it higher than the margins and so on until the cavity is full, thus gaining the valuable qualities of adhesive gold, and the expansive force of nonadhesive when the wedge is used, as well as what there may be of expansion of the gold by mere pressure, which has been made directly downward with large and small points.

The cavity being filled is finished as before described.

The *approximal surfaces* of these teeth after separation are subject to the same general rules in filling, except that pressure is made in the use of both kinds of gold towards the cervical wall, at least until there shall be so much gold in the cavity as that by the approach towards each other of the palatine and labial walls, the gold shall maintain, by its adjustment, the pressure it has received against the cervical walls, when, if using adhesive gold, pressure should be made in the direction of the bottom of the cavity. In the separation of these teeth, the file, enamel chisel, quick or slow pressure, or a combination of several of the methods, as may be suggested by the nature of the case, is or are used. If space is taken with the file or chisel the greatest aperture should generally be on the palatine surface, and sufficient for convenient approach to the several parts of the cavity. So much might be said of the different methods of separating teeth, that we do not feel called upon to say more about it, except that we should never carry the separation so far as to pass the points of contact, by which means they would again approximate, thus rendering a return of decay almost certain.

It often happens that the *anterior surface of the second molar* is a seat of decay, in consequence of the decay of the first molar, which is formed and erupted at a period in life when there is so generally present a disorder in the functions of the body. In January 1870, we were called on to operate for a young lady living in the South and aged 22 years. After treating some ten or fifteen carious openings in her teeth, we examined the anterior approximal surface of the first superior molar on the left side and found that the decay had encroached so much upon the nerve pulp as to uncover at least half of its surface; there were only walls of enamel on the palatine and buccal sides, and about one-fourth of the grinding surface was gone. The pulp was in a perfectly healthy condition, as that also of the surrounding parts, and the patient's health was good. The decay was all carefully removed and the lining membrane not wounded. The cavity was dried and protected from moisture, and then bathed for a few minutes with carbolic acid, when I filled the cavity with *os-artificiel* and evaporated on its surface a drop of liq. gutta percha, when after a space of three or four hours I carved down the filling to the proper distance to enable me to make a good gold one, and filled with Watt's Crystal Gold, with mallet force. No inconvenience has since been felt and the tooth has every appearance of having all of its original vitality.

In June 1870, we operated on the same tooth in the mouth of a younger sister, about 19 or 20 years of age, decayed in the same place and in the same way, except that neither the nerve pulp nor the lining membrane were exposed. The tooth was extremely sensitive and the decay was removed with great difficulty. When prepared for filling it was so extremely painful that the patient could not tolerate the contact of any metallic substance on the bottom of the cavity; and it was so shallow that none of the usually servicable non-conductors could be used. It was bathed for some time in creosote, and then having saturated a small piece of letter paper with creasote, I placed it on the bottom of the cavity and filled with crystal gold, with comfort. This method was pursued because the patient wished to be absent from home for some months, and we did not think that the conditions of the fluids of the mouth would warrant a temporary filling. I had to choose between the loss of the tooth from absorption of a temporary filling, or of inflammation produced by thermal shocks conveyed through a metallic filling. I endeavored to choose a safe course, but was not successful. The patient lost the tooth about one month afterwards, while away from home from severe odontalgia.

I do not yet know what I ought to have done, and I gained neither success or wisdom, unless it be the latter. Hereafter I will pursue an expectant course in such cases, and if the patient does not or cannot return at a proper time, it will not be my fault at least.

